# Stem Cell Applications in Regenerative Medicine for Kidney Diseases

**DOI:** 10.1155/2021/9817324

**Published:** 2021-08-03

**Authors:** Érika B. Rangel, Cláudia O. Rodrigues, Nádia K. Guimarães-Souza

**Affiliations:** ^1^Hospital Israelita Albert Einstein, São Paulo, Brazil; ^2^University of Miami Miller School of Medicine, Miami, USA

Broad clinical conditions can lead to kidney disease, which is a worldwide public health problem that affects millions of people from all sexes, ages, and racial groups. Acute kidney injury (AKI) may be secondary to kidney hypoperfusion, direct kidney damage due to toxins, sepsis, or immune-mediated, and postobstructive. AKI occurs not only in patients without previous kidney impairment but also in a manner of superimposed on the chronic kidney disease (CKD) settings. Overall, CKD is mainly caused by diabetic kidney disease (DKD), hypertension, and glomerulonephritis. Therefore, the search for nonpharmacological strategies for controlling kidney diseases is of utmost importance.

The present special issue has been designed to stimulate continuing efforts to develop novel therapeutic strategies to accelerate AKI recovery and curtail CKD progression. It includes five review articles and two original papers from leading and emerging scientists with diverse expertise and covers three thematic areas: (i) the therapeutic potential of mesenchymal stem cells (MSCs) in acute and chronic kidney diseases; in particular, AKI caused by toxicants and acute ischemia-reperfusion, and CKD secondary to hypertension, systemic lupus erythematosus (SLE), and diabetes mellitus (DM); (ii) therapeutic perspectives of MSCs in peritoneal fibrosis, a clinical condition that can be found in end-stage kidney disease (ESKD); (iii) and the emerging studies of inducible pluripotent stem cells (iPSCs) and their differentiation into kidney cells.

In the paper of the present special issue entitled “Clinical Efficacy and Safety of Mesenchymal Stem Cells for Systemic Lupus Erythematosus,” T. Zhou et al. performed a meta-analysis comprising prospective and retrospective case series and randomized controlled trials of MSC-based therapy in SLE, in particular, lupus nephritis. That condition may progress to CKD. Importantly, their main findings included that MSCs promoted a decrease in proteinuria at 3 and 6 months and SLE activity, as assessed by a lower score of SLEDAI (at 2 and 6 months); although, that therapy did not yield a reduction in serum creatinine. Regarding key aspects of MSC-based therapy, in eight studies, the MSC source was the umbilical cord (UC), whereas two studies included a combination of UC and bone marrow- (BM-) derived MSC injection. The number of injected cells diverged in the studies, from 1 × 10^6^/kg or 5 × 10^7^ to 2 × 10^8^ cells per infusion, as well as the number of injections (1 to 5 times). Except for one study that injected MSCs via the renal artery, the other studies used the intravenous route for cell injection. MSC efficacy may be affected by several conditions not only related to their administration (source, dose, frequency, and route) but also by their heterogeneity due to culture conditions. Therefore, the International Society for Cell Therapy proposed the assessment of MSC functionality based on assays that demonstrate the secretion of trophic factors, modulation of immune cells, and differentiation capacity [1]. Thus, injecting less heterogenic and more functional MSCs will improve clinical outcomes.

In line with the benefits of MSC in CKD setting, a study by E. C. Costalonga et al. entitled “Adipose-Derived Mesenchymal Stem Cells Modulate Fibrosis and Inflammation in the Peritoneal Fibrosis Model Developed in Uremic Rats” described a model of CKD (induced by 0.75% adenine-containing diet for 30 days) and peritoneal fibrosis (induced by chlorhexidine gluconate) in a preclinical model. In ESKD, patients under peritoneal dialysis are at high risk of developing peritoneal fibrosis and losing the efficacy of their treatment [2]. The authors documented that adipose-tissue-derived MSC (1 × 10^6^ cells, 2 times, and intravenous route) abrogated peritoneal fibrosis and fostered antifibrotic (decrease in TGF-*β*, fibronectin, and collagen) and anti-inflammatory (decrease in IL-1*β*, TNF-*α*, and IL-6) responses. Therefore, the results obtained from this study contribute to set the basis for establishing further investigation of the therapeutic potential of MSCs for peritoneal fibrosis treatment in both preclinical and clinical studies.

There is compelling evidence of the therapeutic potential of MSCs for kidney disease, but whether microvesicles (MVs) and exosomes (EXs) released by these cells hold similar efficacy is still a subject of debate in the literature. In the preclinical study by C. S. R. A. Ishiy et al. entitled “Comparison of the Effects of Mesenchymal Stem Cells with Their Extracellular Vesicles on the Treatment of Kidney Damage Induced by Chronic Renal Artery Stenosis,” the authors developed a model of renovascular hypertension (2 kidneys, 1 clip) in rats and compared the outcomes after adipose tissue-derived MSC injection, and MV and EX treatment. All three treatments (adipose tissue-derived MSCs, MVs, and EXs) reduced blood pressure, proteinuria, and the expression of collagen type I and TGF-ß in kidney cortex and medulla when compared to the stenotic kidney and sham, yet not preventing the increase in heart weight, a finding that is secondary to systemic arterial hypertension. In addition, IL-10 levels increased in all three treatments. Of note, adipose tissue-derived MSC and MVs contributed to lower proteinuria values more than the treatment with EXs, whereas whole cells were more effective in reducing the inflammatory cytokine IL-1*β*. The results of this study shed light on the importance of selecting the most appropriate therapy in accordance with the main target of a specific disease.

To further substantiate the investigation of the impact of MSC on functional and structural results in preclinical models using small and large animals and the translational approach to clinical studies, the review of C. Sávio-Silva et al. entitled “Mesenchymal Stem Cell Therapy for Diabetic Kidney Disease: A Review of the Studies Using Syngeneic, Autologous, Allogeneic, and Xenogeneic Cells” addressed the main issues of MSC-based therapy for diabetic kidney disease (DKD). The authors highlighted the importance of recapitulating DKD microenvironment and the interaction of cell-cell and cell-matrix interactions in vitro for evaluating the therapeutic potential of MSCs. They also critically analyzed the main challenges of evaluating MSC efficacy in preclinical models, such as MSC phenotype, source, route of delivery, and homing. Notably, there are several rodent models of type 1 and type 2 DM that can be used to verify the MSC therapeutic potential and assessment of functional and structural outcomes in kidney disease. Taking a step forward, the authors documented the main findings of clinical trials using autologous or allogenic-derived MSCs for type 1 and type 2 diabetic individuals. Noteworthily, MSC-based preclinical and phase I/II clinical data encourage the design of future large-scale controlled clinical trials that evaluate DKD response to MSC therapy; although, type 2 DM-derived MSCs raised some concerns about their efficacy due to changes in their microenvironment imposed by hyperglycaemia. Importantly, assessing donor-to-donor MSC heterogeneity, the potential immunogenicity of allogenic-derived MSCs, and their differentiation state should be taken into account in future studies [3, 4].

In the manuscript entitled “The Efficacy of Mesenchymal Stem Cells in Therapy of Acute Kidney Injury Induced by Ischemia-Reperfusion in Animal Models,” T. Zhou et al. discussed at length the main findings of MSC-based therapy in AKI setting. In their meta-analysis comprising acute ischemia-reperfusion injury, MSC promoted a decrease in creatinine levels at short- (1 day) and long- (>7 days-) terms, as well as in proteinuria. Increased creatinine and proteinuria are two crucial hallmarks of kidney dysfunction and may predict progression to CKD [5]. Importantly, MSC treatment specifically decreased AKI-related targets, such as markers of oxidative stress, inflammation, and fibrosis, which ultimately lead to structural damage amelioration. Therefore, we can anticipate that MSC-based therapy may prevent AKI progression to CKD and may mitigate the damage of AKI superimposed on CKD.

In line with the findings of MSC-based therapy in AKI, S. Lin et al. performed a meta-analysis entitled “Nephroprotective Effect of Mesenchymal Stem Cell-Based Therapy of Kidney Disease Induced by Toxicants” and verified the efficacy of MSC-based treatment in AKI injury secondary to diverse toxicants (glycerol, cisplatin, adriamycin, methotrexate, streptozotocin, cadmium, rifampicin, gentamicin, and aristolochic acid). Despite different sources (bone marrow, adipose tissue, umbilical cord, amniotic fluid, and embryonic stem cells), routes (intravenous, intraperitoneal, intra-aorta, subcapsular, intrarenal parenchyma, and subcutaneous), and doses (2 × 10^5^ − 5 × 10^6^) that were analyzed, MSC treatment decreased serum creatinine, serum blood urea nitrogen, and albuminuria levels, as well as restored the imbalance of prooxidants and antioxidants within the kidneys, and ameliorated inflammation and fibrosis. These findings paved the way for the development of clinical trials in a broad AKI setting.

To gain further insight into the novel strategies to repair kidney damage and improve our understanding of signaling pathways involved in renal homeostasis and injury, in the manuscript entitled “Differentiating Induced Pluripotent Stem Cells into Renal Cells: A New Approach to Treat Kidney Diseases,” P. de Carvalho Ribeiro et al. discussed the main protocols available in the literature for the differentiation of IPSCs into renal cells from human and rodent sources. These protocols may take from 4 to 26 days to be performed and set the basis for fostering the development of renal progenitors and podocyte or tubular-like cells that can be injected into animals to repair kidney damage. Notably, this knowledge also contributed to the advancement of developing kidney organoids for regenerative medicine.

In conclusion, the bench to bedside pathway has been constructed for MSC-based treatment in the kidney disease setting. Experimental animal models indicated that MSCs are effective for treating acute and chronic kidney diseases. MSCs demonstrated efficacy in controlling several biological processes, such as oxidative stress, inflammation, and fibrosis, as well as in ameliorating renal functional and structural parameters. Therefore, it is important to comprehend and interpret these experimental data and equally important to critically review clinical studies. Despite the data encouraging the design of controlled randomized clinical trials to evaluate acute and chronic kidney disease response to MSC-based therapy, rigorous reporting of safety and efficacy is still needed.
